# Traumatologie – eine interdisziplinäre Aufgabe: nur in der Lehre?

**DOI:** 10.1007/s00106-022-01255-w

**Published:** 2022-12-16

**Authors:** C. Offergeld, B. Hofauer, P. Poxleitner, W. Lagrèze, O. Schnell, N. Petersen, F. Lang, V. Burkhardt, J. Pfeiffer, T. Albrecht

**Affiliations:** 1 Univ.-HNO-Klinik, Medizinische Fakultät, Universitätsklinikum Freiburg, Killianstr. 5, 79106 Freiburg, Deutschland; 2 Univ.-HNO-Klinik, Universitätsklinikum TUM München, München, Deutschland; 3 Univ.-Klinik für MKG-Chirurgie, Universitätsklinikum Freiburg, Freiburg, Deutschland; 4 Univ.-Augenklinik, Universitätsklinikum Freiburg, Freiburg, Deutschland; 5 Neurochirurgische Univ.-Klinik, Universitätsklinikum Freiburg, Freiburg, Deutschland; 6 Studiendekanat, Universitätsklinikum Freiburg, Freiburg, Deutschland; 7 Univ.-HNO-Klinik, Universitätsklinikum Freiburg, Freiburg, Deutschland; 8 HNO-Praxis am Theater, Freiburg, Deutschland; 9 Univ.-HNO-Klinik, Universitätsklinikum Tübingen, Tübingen, Deutschland

**Keywords:** Traumatologie, Interdisziplinäre Vermittlung, Berufliche Ausbildung, Patientenversorgung, Online-Lehre, Traumatology, Interdisciplinary placement, Professional education, Patient care, Online learning

## Abstract

**Hintergrund:**

Die Traumatologie des Kopf-Hals-Bereichs stellt nicht nur einen Teilbereich des HNO-Fachgebiets dar, sondern bildet mit ihren Krankheitsbildern zudem eine große Schnittmenge mit Nachbardisziplinen der Kopf-Hals-Region. In Freiburg wurde im Wintersemester 2021/2022 eine interdisziplinäre Vorlesung zu „HNO-Notfällen“ implementiert. Ziel war ein realistischerer Bezug zur interdisziplinären Patientenversorgung und die Kenntlichmachung von Schnittmengenbereichen von 4 der wesentlichen Kopfdisziplinen (HNO-Heilkunde, Neurochirurgie, Augenheilkunde, Mund-Kiefer-Gesichts[MKG]-Chirurgie).

**Material und Methoden:**

Im Rahmen der regulären, semesterbegleitenden Online-Vorlesungsreihe wurde eine neue, spezielle Vorlesung in der HNO-Lehre implementiert. Mit Bezug auf die klinische Versorgung von HNO-Notfällen wurden mögliche Überschneidungen mit Nachbardisziplinen ausgewiesen und von den jeweiligen Fachvertretern erläutert bzw. vor und mit dem Auditorium diskutiert. Zum Semesterende wurde diese Lehrveranstaltung mittels des Befragungstools „EvaSys“ (Fa. EvaSys GmbH, Lüneburg, Deutschland) für alle partizipierenden Studierenden (*n* = 173) freigegeben. Es beteiligten sich 78 Studierende an der Evaluation.

**Ergebnisse:**

Das neue Vorlesungskonzept wurde außerordentlich gut akzeptiert und auf Anhieb auf die Top-Position der interdisziplinären Veranstaltungen aus der HNO-Vorlesungsserie gewählt. Auch die anschauliche Vermittlung des Begriffs „Interdisziplinarität“ im Sinne einer sich ergänzenden klinischen Fächerkooperation gelang sehr erfolgreich und wurde von den Studierenden entsprechend bei der Evaluation gewürdigt.

**Schlussfolgerung:**

Die HNO-Lehre erlaubt die pragmatische Darstellung einer idealen klinischen Patientenversorgung mithilfe eines interdisziplinären Ansatzes. Diese realistische Darstellung, jenseits jeglicher fachlicher und/oder berufspolitischer Differenzen, ist für die Studierenden von großem Interesse und wird als klinisch relevant eingestuft. Damit bietet die Lehre eine wertvolle Möglichkeit, die wesentlichen Werte einer klinischen interdisziplinären Versorgung im Sinne der bestmöglichen Patientenversorgung zu vermitteln.

## Abstract (en)

**Background:**

Traumatology of the head and neck region is not only a part of otorhinolaryngology, but also has a large overlap with neighboring disciplines of the head and neck region. In Freiburg, an interdisciplinary lecture on “ENT emergencies” was implemented in the 21/22 winter semester. The aim was to provide an even more realistic view on interdisciplinary patient care and to make evident the areas of intersection of four of the major head disciplines (otorhinolaryngology, neurosurgery, ophthalmology, and maxillofacial surgery).

**Materials and methods:**

A new, special lecture in otorhinolaryngology was implemented as part of the regular online lecture series accompanying the semester. With reference to the clinical care of ENT emergencies, possible overlaps with neighboring disciplines were identified and explained by the discipline representatives or discussed in front of and with the auditorium. At the end of the semester, all participating students (*n* = 173) were invited to evaluate the seminar using the survey tool “EvaSys” (EvaSys GmbH, Lüneburg, Germany). In total, 78 students participated in the evaluation process.

**Results:**

The new lecture concept was very well accepted and immediately ranked top among the interdisciplinary lecture titles within the ENT lecture series. The clear communication of the term “interdisciplinarity” in the sense of a complementary clinical cooperation was also very successful and was appreciated accordingly by students during the evaluation process.

**Conclusion:**

Pragmatic presentation of ideal clinical patient care using an interdisciplinary approach is possible within the regular ENT lecture series. This realistic portrayal, beyond any technical and/or professional differences, is of great interest to students and is considered clinically relevant. Thus, interdisciplinary lectures provide a valuable tool to teach the fundamental values of clinical interdisciplinary management for the best possible patient care.

Die Traumatologie stellt einen Teilbereich der HNO-Weiterbildung dar. Dabei umfasst die Traumatologie im Kopf-Hals-Bereich sowohl Verletzungen der Schädelbasis, der zentrolateralen Mittelgesichtsregion, der Orbita, des Schädels und des Halses. Der immense Umfang an Krankheitsbildern einer komplexen anatomischen Region hat mit zunehmender Expertise der medizinischen Fachdisziplinen ein gewisses Maß an Subspezialisierung erfahren [2]. Folglich haben die einzelnen Fachgesellschaften mitunter diagnostische und therapeutische Verfahren erprobt und entwickelt, welche eine fachbezogene Abgrenzung erlauben können. Zudem existieren fachliche Schnittmengenbereiche, welche jedoch weitgehend diskussionslos einer Disziplin primär zugeordnet werden dürfen (z. B. Laterobasis und HNO-Heilkunde) ohne allerdings dabei den Anspruch auf absolute Exklusivität in Anspruch nehmen zu können.

## Problembereich klinischer Alltag

Wenngleich es tatsächlich Bereiche der friedlichen Koexistenz im Kopf-Hals-Bereich gibt, existieren auch Überschneidungsbereiche zwischen den einzelnen Fachdisziplinen, wo die grundlegenden Vorgehensweisen keine nennenswerten Unterschiede erwarten lassen, aber – einer zugrunde liegenden „Philosophie“ folgend – in betont unterschiedlicher Weise (z. B. chirurgischer Zugangsweg) durchgeführt werden. Somit kann es im klinischen Alltag einer Notaufnahmeeinheit mitunter zu Unstimmigkeiten zwischen den Fachgebieten bezüglich der Versorgungshoheit bzw. des diagnostischen und insbesondere therapeutischen Prozedere von akut verunfallten Patienten kommen. Dies geschieht oftmals in Abhängigkeit von der Verletzungslokalisation (z. B. Orbita/Mittelgesicht) und der sich zuständig fühlenden Klinik. Dabei nehmen die jeweiligen Disziplinen (evidenzbasierte) Aussagen und/oder Erfahrungswerte für sich in Anspruch, welche in unterschiedlicher Qualität den Führungsanspruch belegen sollen [3]. Hier kann es zu grundsätzlichen, z. T. sogar erbitterten Auseinandersetzungen zwischen einzelnen Klinikvertreter:innen kommen. Tatsächlich existieren mitunter deutliche Unterschiede in Quantität und Qualität der wissenschaftlichen Publikationstätigkeit bezüglich traumatologischer Fragestellungen, bei welchen die HNO-Heilkunde nicht gerade eine Spitzenposition einnimmt [11].

## Wahrnehmung in der Lehre

Die jährlichen Evaluationen der Studierenden in der HNO-Lehre haben wiederholt den Wunsch nach Änderungen des Lehrbetriebs aufgezeigt. Unter diversen Maßnahmen hatte die HNO-Lehre sich primär der traditionellen Vorlesung angenommen. Das „3D-Format“ mit Darstellung der Lehrinhalte dreier komplexer anatomischer Regionen (Mittelohr, Nase/Nasennebenhöhlen [NNH] und Larynx) aus 3 Blickwinkeln (HNO-Heilkunde, Radiologie und Anatomie) wurde positiv aufgenommen, ohne jedoch nachweislich eine Top-Ranking-Position innerhalb der eigenen Vorlesungsreihe zu belegen [1]. Gleichwohl wurde des Öfteren in Freitextkommentaren der Wunsch nach mehr klinischer Relevanz der Vorlesungsinhalte und der Einbeziehung von Nachbardisziplinen geäußert. Somit wurde im Sommersemester 2020 erstmalig eine interdisziplinäre Vorlesung in der HNO-3D-Reihe pilotiert. Die positiven studentischen Rückmeldungen (trotz rein virtueller Präsentation) veranlassten das Team der HNO-Lehre Freiburg, diesem Format weiter nachzugehen. Unter dem Fokus „HNO-Notfälle“ wurde die Vorlesung interdisziplinär unter Beteiligung der Fachgebiete Neurochirurgie, Augenheilkunde, Mund-Kiefer-Gesichts(MKG)-Chirurgie und HNO-Heilkunde ausgerichtet und als interaktives Lehrformat angeboten. Ziel war die Präsentation von 4 wesentlichen Kopfdisziplinen und deren Möglichkeiten zur interdisziplinären Zusammenarbeit bei der klinischen Versorgung von Notfallpatient:innen. Gleichzeitig konnten sich die einzelnen Fachgebiete präsentieren und ihre klinischen Schwer- sowie Standpunkte veranschaulichen.

## Material und Methoden

Im Rahmen des HNO-Blockpraktikums wurde semesterbegleitend eine Vorlesungsreihe angeboten. Innerhalb dieser wurde ein Extracurriculum mit 3 „3D-Vorlesungen“ ausgewiesen. In diesem Segment konnte dann die neue interdisziplinäre Vorlesung „HNO-Notfälle“ zuerst pilotiert und dann sukzessive implementiert werden. Im Wintersemester 21/22 hatten insgesamt 173 Studierende die Möglichkeit, an dieser Vorlesung teilzunehmen. Alle Teilnehmer wurden schriftlich aufgefordert, an der Fokusevaluation zu partizipieren. Ausgewertet wurden 8 Fragen zur HNO-3D-Vorlesungsreihe sowie 4 Fragen zur Interdisziplinarität. Zusätzlich wurde von den Studierenden ein persönliches Ranking der 4 3D-Vorlesungen erbeten. Die Auswertung der Evaluationsergebnisse erfolgte unter Beachtung von Standardabweichung und Mittelwert durch Verwendung des Systems „EvaSys“ (Fa. EvaSys GmbH, Lüneburg, Deutschland) in Kooperation mit dem Studiendekanat der Medizinischen Fakultät Freiburg. Dieses System ist eine webbasierte Plattform und dient der Automation von Befragungen. Abgebildet wurde die prozentuale Verteilung der Antworten (relative Häufigkeit) auf einer 6‑stufigen Likert-Skala bzw. die exportierten Mittelwerte in tabellarischer Form. Die statistische Erhebung und Auswertung der nichtvalidierten Fragebögen erfolgte über das Studiendekanat der Medizinischen Fakultät des Universitätsklinikums Freiburg.

## Ergebnisse

**Figure 1:**
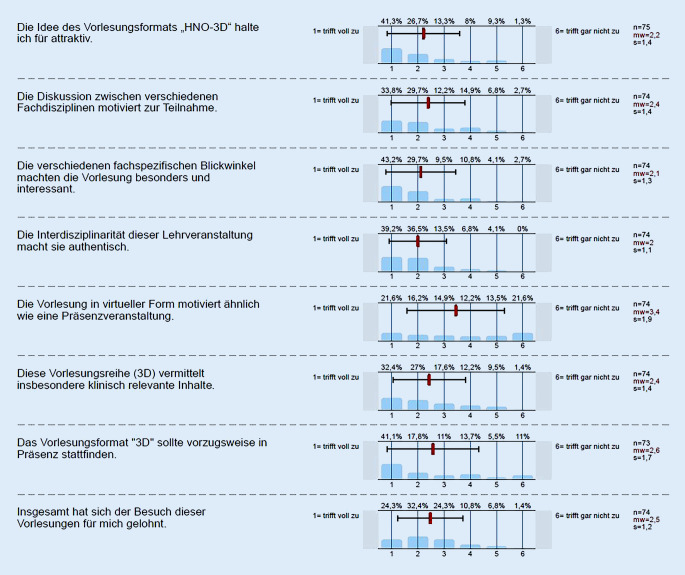

**Figure 2:**
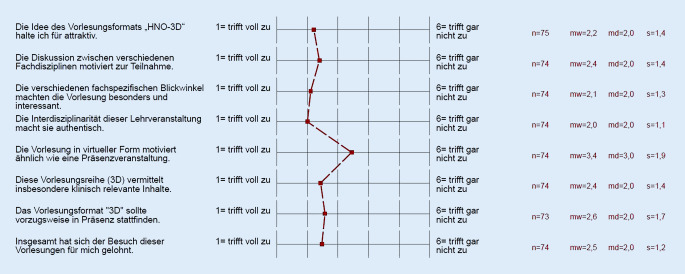

Es nahmen 78 Studierende an der Befragung teil bzw. sandten ihre adäquat ausgefüllten Fragebögen zurück. Die Erfassung von 78 Fragebögen entspricht einer prozentualen Rücklaufquote von 45,6 %. Die Auswertung zeigt die Verteilung der Antworten auf einer 6‑stufigen Likert-Skala, wobei eine „1“ einer vollständigen Zustimmung entspricht und eine „6“ einer kompletten Ablehnung. Wie in Abb. 1 ist sowohl die relative prozentuale Verteilung als auch der Mittelwert für jede einzelne Frage dargestellt. Zusätzlich sind die Mittelwerte in einer Übersicht gesondert als Profillinie dargestellt (Abb. 2). Die Auswertung fokussierte auf 2 Parameter:

### HNO-3D-Vorlesungsreihe

**Figure 3:**
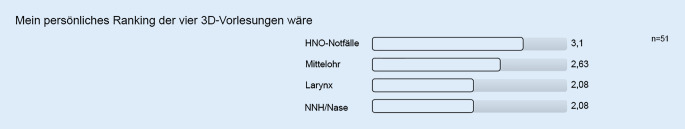

Die Konzeption des HNO-Vorlesungsformats wurde mehrheitlich positiv (62 %) aufgefasst (Abb. 1). Der interdisziplinäre Aspekt wurde von einer klaren Mehrheit der Studierenden unter verschiedenen Fragestellungen als motivierend, authentisch, klinisch relevant und interessant eingestuft (Likert 1: bis zu 43,2 % komplette Zustimmung; Abb. 1 und 2). Zudem waren die Studierenden mehrheitlich (Likert 1, Likert 2: 56,7 % Zustimmung, Abb. 1 und 2) der Auffassung, dass ihnen ein Vorteil durch die Teilnahme entstanden sei. Gleichwohl votierten die Teilnehmer zu annähernd gleichen Teilen positiv wie negativ auf die Frage, ob eine Präsenzveranstaltung zu favorisieren wäre (Abb. 1: Likert 1, Likert 2: bis zu 37,8 % vs. Likert 5, Likert 6: bis zu 35,1 %). Somit konnte keine eindeutige Gleichwertigkeit von Präsenz- und virtueller Lehrveranstaltung nach Ansicht der Studierenden festgestellt werden. Dies zeigt sich auch bei der Frage nach Vorzug einer Präsenzvariante gegenüber der Online-Version. Hier wurde eindrücklich auf die Präsenzveranstaltung (Likert 1, Likert 2: bis zu 58,9 %) verwiesen (Abb. 1). Im Ranking der vier 3D-Vorlesungen (Abb. 3) wurde die interdisziplinäre Vorlesung „HNO-Notfälle“ auf Anhieb auf den ersten Platz gewählt. Dahinter platzierten sich die Vorlesungen Mittelohr, Larynx und NNH.

### Interdisziplinarität

**Figure 4:**
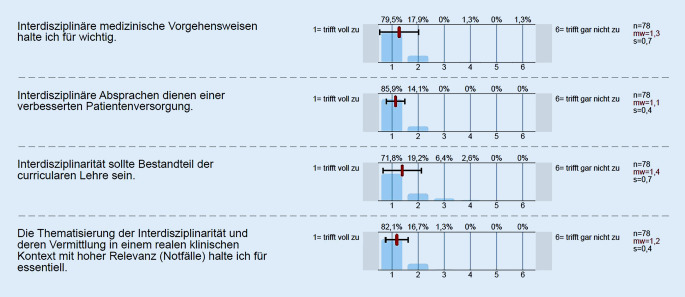

Die evaluierenden Studierenden waren ausnahmslos der Ansicht, dass Interdisziplinarität ein hohes Gut darstellt und einer idealen Patient:innenversorgung zugeordnet werden kann (Abb. 4). Dies wurde von den evaluierenden Studierenden ausdrücklich mit hoher Relevanz für den klinischen Einsatz (Patient:innenversorgung) festgestellt. Der überwiegende Teil der befragten Teilnehmer:innen sah sogar die Thematik als essenziellen Bestandteil der curricularen Lehre an (71,8 % Likert 1; Abb. 4) bzw. empfand die Möglichkeit der Abbildung innerhalb der curricularen Lehre als attraktiv. Ebenso deutlich fiel die Evaluation für die Thematisierung der Interdisziplinarität (82,1 % Likert 1; Abb. 4) in der vorliegenden Erhebung aus.

## Diskussion

Die Fachgebiete der Kopf-Hals-Region sind partiell subspezialisierte Disziplinen, welche sich einerseits ergänzen können, aber andererseits einen gewissen „Exklusivitätsaspekt“ für sich beanspruchen. Dieses Sendungsbewusstsein ist ubiquitär in unterschiedlichem Ausmaß ausgeprägt. Im Zuge der organisatorischen Zusammenführung dieser sich z. T. in Konkurrenz befindlichen Fachdisziplinen gab es innovative Ansätze wie z. B. die bauliche Vereinigung der Kopfdisziplinen in „Kopfkliniken“ in Würzburg oder Heidelberg bereits in den 1980er-Jahren.

Aber auch aktuell gibt es berufspolitische Entwicklungen, die zum Nachdenken anregen. Hier ist beispielsweise ein nationaler bzw. europäischer Disput – vorrangig um Zuordnung von Weiterbildungsinhalten – zu verzeichnen, welcher aber auch Auswirkungen auf die curriculare Lehre haben kann. Dies vor dem Hintergrund, dass die Musterweiterbildungsordnung (MWBO) im Idealfall eine Abbildung von Kompetenzen vorsieht. Momentan scheint dies aber eher ein hehres Ziel denn eine zielführende und effektive Implementierung zu sein. Im Bereich der Ausbildung hingegen ist die Implementierung des Nationalen Kompetenzbasierten Katalogs in der Medizin (NKLM) bereits Realität. Dieses Katalogwerk richtet sich nicht nach Fachdisziplinen, sondern nach Organsystemen und fordert eine Kompetenzorientierung in interdisziplinärer Form. Somit besteht hier durch die Lehre eine große Chance, die Vorteile eines tatsächlichen interdisziplinären Konzepts aufzuzeigen und womöglich positiv zu beeinflussen.

### Traumatologische Entwicklungen

Die vergangenen Dekaden seit der Jahrtausendwende haben eine wechselhafte Entwicklung des Teilbereichs Traumatologie in der HNO-Heilkunde mit sich gebracht. Gerade in der chirurgischen Therapie von Mittelgesichtsverletzungen konnte man diagnostische und therapeutische Weiterentwicklungen verzeichnen. So wurde beispielsweise der diagnostische Wert der Sonographie bei Mittelgesichtsfrakturen oder auch deren intraoperativer Einsatz von mehreren Fachgebieten bearbeitet und publiziert, u. a. von Vertreter:innen der HNO-Heilkunde [4, 6–8].

Etwas anders zeigt sich die Entwicklung bei neuen Ansätzen zur chirurgischen Versorgung von Mittelgesichtsfrakturen. Hier ist die Entwicklung an der HNO-Heilkunde weitgehend vorbeigegangen [5]. Diese und andere Umstände (s. Editorial zu diesem Leitthema) haben zu Anspruchshaltungen und Meinungsdiversitäten zwischen den verschiedenen Fachdisziplinen geführt und/oder unterhalten diese immer noch. Derartige Differenzen stehen aber einer idealen interdisziplinären Versorgung im Sinne der Lehrmeinung diametral entgegen und sollten einer modernen Interdisziplinarität nicht im Wege stehen.

### Lehre und Lernziele

Die moderne Lehre orientiert sich an der Lehrforschung und reflektiert aktiv das aktuelle Meinungsbild der Adressaten und integriert es u. U. sogar [13]. Dies erlaubt eine Weiterentwicklung des Lehrkonzepts auf der Basis einer pragmatischen Analyse vorgenommener Veränderungen ohne Rücksicht auf Befindlichkeiten des klinischen Alltags. Im vorliegenden Fall wurde dem Wunsch einer interdisziplinären Vorlesung entsprochen. Dies geschah ohne Rücksicht auf vorher gemachte Erfahrungen im gleichen Segment der 3D-Vorlesungen [1]. Im Gegensatz zu den Ergebnissen von Daubenfeld et al. wurde die neue Vorlesung bei ihrer Implementierung auf Platz 1 der 3D-Vorlesungen gewählt (Abb. 3). Die Evaluation der Studierenden zeigt eindeutig die Wertschätzung der klinischen Relevanz und des erfolgreichen „Exports“ klinischer Entscheidungspfade in das sonst eher abstrakt-theoretische Vorlesungsformat. Die Stringenz und professionelle Präsentation der jeweiligen Fachvertreter:innen hatten dabei einen entscheidenden Anteil an einer durch die Studierenden als authentische, motivierende und interessant wahrgenommenen Lehrveranstaltung. Somit konnte unter Aussparung möglicher differenter Sichtweisen der Prozess einer idealen Patientenversorgung im interdisziplinären Setting erfolgreich vermittelt werden.

Das Erfüllen der Lernziele zeigte sich auch an der Evaluation der Begrifflichkeit „Interdisziplinarität“. So wurde von einer überwiegenden Mehrheit der Evaluierenden eine interdisziplinäre Vorgehensweise als wichtig erachtet (Abb. 4). Zudem wurde nach Ansicht der Studierenden eine verbesserte Patient:innenversorgung an die Möglichkeit interdisziplinärer Absprachen gekoppelt. Ebenso eindrucksvoll ist die Einschätzung der Notwendigkeit einer fächerübergreifenden Absprache zur bestmöglichen und effektivsten Patient:innenversorgung, insbesondere im Notfallsetting. Hier scheinen effektive Zeitkorridore und stringente Vorgaben, wie beim Management traumatologischer Akutpatienten in der Rettungsstelle/Notaufnahme, die treibenden Vorstellungen zu generieren [12]. Auffällig ist die studentische Einschätzung der virtuellen gegenüber der Präsenzlehre. Trotz aller Fortschritte und deutlichen Zuspruchs für virtuelle Konzepte [14] gibt es in der vorliegenden Erhebung ein annähernd ausgewogenes Verhältnis zwischen Befürworter:innen und Gegner:innen bezüglich der durch die Veranstaltung ausgelösten Motivation und eine Präferenz für das Präsenzformat (Abb. 1). Hier zeigt sich auch die partiell widersprüchliche Situation der (studentischen) Evaluation. Es wird dem Lehrformat einerseits Attraktivität und Interesse auslösende Wirkung attestiert, andererseits liegen die persönlichen Zustimmungswerte („lohnenswert“) deutlich darunter (Abb. 1). Ebenso verhält es sich beim Zuspruch für die diversen Diskussionsstandpunkte der Fachdisziplinen und der empfundenen klinischen Relevanz dieser Lehrveranstaltung. Wenngleich in beiden Punkten eine deutliche Mehrheit diese Aussagen befürwortet, so zeigen sich immerhin 15 % respektive annähernd 22 % der Evaluierenden kritisch (Abb. 1). Da aber alle Aussagen (mit Ausnahme der Frage nach vergleichender Motivation durch eine Präsenzveranstaltung) insgesamt mit einer deutlichen Mehrheit (> 50 % Likert 1 und Likert 2; Abb. 1) positiv evaluiert wurden, weist dies auf einen paradoxen Effekt bei Evaluationen hin, welcher Beachtung verdient, aber nicht überbewertet werden sollte und nicht immer rational erklärt werden kann. Derartige Phänomene sind aus verschiedenen Erhebungen bekannt, z. B. aktuell aus der virtuellen Lehre [14]. Demgegenüber besonders beeindruckend ist hingegen die annähernd einhellige studentische Meinung, dass der Begriff und mögliche praktische Auswirkungen der Interdisziplinarität bereits in der curricularen Lehre verankert sein sollten. Eine durchaus vorstellbare Konstellation, welche einer patientenzentrierten Ausbildung sicher zuträglich wäre.

Man wird abwarten müssen, inwiefern der NKLM 2.0 bereits die Chancen bei der Entwicklung interdisziplinärer Veranstaltungen erhöhen kann. Momentan ist der NKLM 2.0 noch ein vergleichsweise rigides Katalogwerk, welches eine Kartierung von Lernzielen im Sinne der Arbeitsgemeinschaft der Wissenschaftlichen Medizinischen Fachgesellschaften e. V. (AWMF), die dazu eine Fächerempfehlung gegeben hat, (noch) nicht zulässt. Es ist aber durchaus vorstellbar, dass zukünftig die Struktur des NKLM zum Mapping einzelner Lernziele fachspezifisch genutzt werden kann. Diese Perspektive ist auch der Medizinischen Fakultät der Universität Freiburg bewusst, die das Projekt positiv begleitet hat. Zudem konnte eine weitere Verbesserung der ohnehin guten Kommunikation durch das Bindeglied einer interdisziplinären Lehrveranstaltung zwischen den Fachdisziplinen beobachtet werden.

## Ausblick

Es wurde nicht nur ein erfolgreiches Lehrformat mit möglichen Implikationen für die curriculare Lehre implementiert, sondern auch die Möglichkeit einer pragmatischen Effektivität für die traumatologische Patientenversorgung unter Auslassung fachspezifischer Differenzen aufgezeigt. Diese Synergien zwischen den Fachgebieten sollten daher im besten Sinne der curricularen Ausbildung und postcurricularen Weiterbildung im klinischen Alltag genutzt und im Lehrbetrieb authentisch vermittelt werden [9, 10].

## Fazit für die Praxis

- Der Vorstoß der HNO-Lehre hat ein vergleichsweise unerwartetes, aber sehr zufriedenstellendes Ergebnis zutage gefördert.
- Zum einen wurde ein erfolgreiches Lehrformat mit möglichen Implikationen für die curriculare Lehre implementiert, zum anderen ergab sich die Möglichkeit, sich unter Auslassung fachspezifischer Differenzen pragmatisch für die traumatologische Patientenversorgung einzusetzen.
- Diese Synergien zwischen den Fachgebieten sollten im klinischen Alltag genutzt und im Lehrbetrieb authentisch vermittelt werden.
- Hierfür könnte der Nationale Kompetenzbasierte Katalog in der Medizin (NKLM) zukünftig einen probaten Katalysator darstellen.
- Eine Umsetzung wie im Freiburger Projekt beinhaltet die Möglichkeit, die Lehre ganz nah an die klinische Realität zu bringen und sowohl die Aus- als auch die Weiterbildung in diesem Segment für alle Disziplinen funktionstüchtig zu halten.
- Dies würde einer „gelebten“ Interdisziplinarität entsprechen, nicht nur in der Lehre.
